# Limbic-predominant age-related TDP-43 encephalopathy (LATE-NC): Co-pathologies and genetic risk factors provide clues about pathogenesis

**DOI:** 10.1093/jnen/nlae032

**Published:** 2024-04-13

**Authors:** Peter T Nelson, David W Fardo, Xian Wu, Khine Zin Aung, Matthew D Cykowski, Yuriko Katsumata

**Affiliations:** Department of Pathology and Laboratory Medicine, University of Kentucky, Lexington, Kentucky, USA; Department of Sanders-Brown Center on Aging, University of Kentucky, Lexington, Kentucky, USA; Department of Sanders-Brown Center on Aging, University of Kentucky, Lexington, Kentucky, USA; Department of Biostatistics, University of Kentucky, Lexington, Kentucky, USA; Department of Sanders-Brown Center on Aging, University of Kentucky, Lexington, Kentucky, USA; Department of Biostatistics, University of Kentucky, Lexington, Kentucky, USA; Department of Sanders-Brown Center on Aging, University of Kentucky, Lexington, Kentucky, USA; Department of Biostatistics, University of Kentucky, Lexington, Kentucky, USA; Department of Pathology and Genomic Medicine, Houston Methodist Hospital, Houston, Texas, USA; Department of Sanders-Brown Center on Aging, University of Kentucky, Lexington, Kentucky, USA; Department of Biostatistics, University of Kentucky, Lexington, Kentucky, USA

**Keywords:** Alzheimer disease neuropathologic change (ADNC), CTE, Carboxy-terminal fragment (CTF), Genome-wide association study (GWAS), Hippocampal sclerosis of aging (HS-A), Frontotemporal lobar degeneration (FTLD), Sulfonylurea receptor type 2 (SUR2)

## Abstract

Limbic-predominant age-related TDP-43 encephalopathy neuropathologic change (LATE-NC) is detectable at autopsy in more than one-third of people beyond age 85 years and is robustly associated with dementia independent of other pathologies. Although LATE-NC has a large impact on public health, there remain uncertainties about the underlying biologic mechanisms. Here, we review the literature from human studies that may shed light on pathogenetic mechanisms. It is increasingly clear that certain combinations of pathologic changes tend to coexist in aging brains. Although “pure” LATE-NC is not rare, LATE-NC often coexists in the same brains with Alzheimer disease neuropathologic change, brain arteriolosclerosis, hippocampal sclerosis of aging, and/or age-related tau astrogliopathy (ARTAG). The patterns of pathologic comorbidities provide circumstantial evidence of mechanistic interactions (“synergies”) between the pathologies, and also suggest common upstream influences. As to primary mediators of vulnerability to neuropathologic changes, genetics may play key roles. Genes associated with LATE-NC include *TMEM106B*, *GRN*, *APOE*, *SORL1*, *ABCC9*, and others. Although the anatomic distribution of TDP-43 pathology defines the condition, important cofactors for LATE-NC may include Tau pathology, endolysosomal pathways, and blood-brain barrier dysfunction. A review of the human phenomenology offers insights into disease-driving mechanisms, and may provide clues for diagnostic and therapeutic targets.

## INTRODUCTION

Limbic-predominant age-related TDP-43 encephalopathy neuropathologic change (LATE-NC) is associated with a clinical syndrome that mimics Alzheimer disease (AD)-type dementia (1). Since the term LATE-NC was introduced in 2019, researchers around the world have found evidence of the large public health impact of the condition. Approximately 1 in 3 individuals beyond age 85 have LATE-NC at autopsy (2). When antemortem cognitive status and other brain pathologies are factored in together, LATE-NC is independently associated with substantial cognitive impairment (3–8). Recent reviews address clinical features of LATE/LATE-NC (9, 10).

Despite progress in the field, uncertainties remain about the biologic mechanisms underlying LATE-NC (10). *Homo sapiens* appear to have unique susceptibility to LATE-NC, and no model system has been developed for specific experimental study of LATE-NC pathogenesis. Therefore, our understanding of LATE-NC is at least preliminarily dependent on piecing together the relevant human phenomenology. Here, we discuss some of the data that have been gathered related to copathologies and genetic risk factors.

Several underlying factors are important to consider although they are not focal points of the present review: (1) the possible influences of chronological aging, for example senescence; (2) the tendency, particularly in persons of advanced age, for brains to harbor multiple pathologies in the same individual; and (3) challenges inherent to understanding how different pathogenetic factors inter-relate, when each individual topic under discussion is an incompletely understood component of a fast-evolving scientific field. Unlike some other neurodegenerative conditions, LATE-NC is most prevalent among the “oldest-old,” and senescence entails many person-specific influences (1, 11–15). Characteristics of aging-related pathologies include both complexity in an individual and heterogeneity across human populations (16–19). The pathologies may affect one another if present in the same brain; as described below, the subcomponents in “mixed” pathologic subtypes may have characteristics that are subtly different from those of “pure” subtypes. The science cultural context is also relevant; research on dementia, as well as its terminology and classification, are in constant flux.

Despite these caveats, the available pathologic and genetic data may provide insights into potential diagnostic biomarkers and therapeutic targets. The number of identified neuropathologic entities associated with dementia is ever increasing although there are overlapping pathologic mechanisms. This prompts hope that (as in other disease paradigms), a future disease-altering remedy that helps for 1 condition may also help for another. This article is written with an overall goal of reviewing the literature of human studies to generate hypotheses about cause-effect relationships in LATE-NC and associated conditions.

## LATE-NC AND COMORBIDITIES THAT ARE ENRICHED IN BRAINS WITH LATE-NC

**Figure 1.**
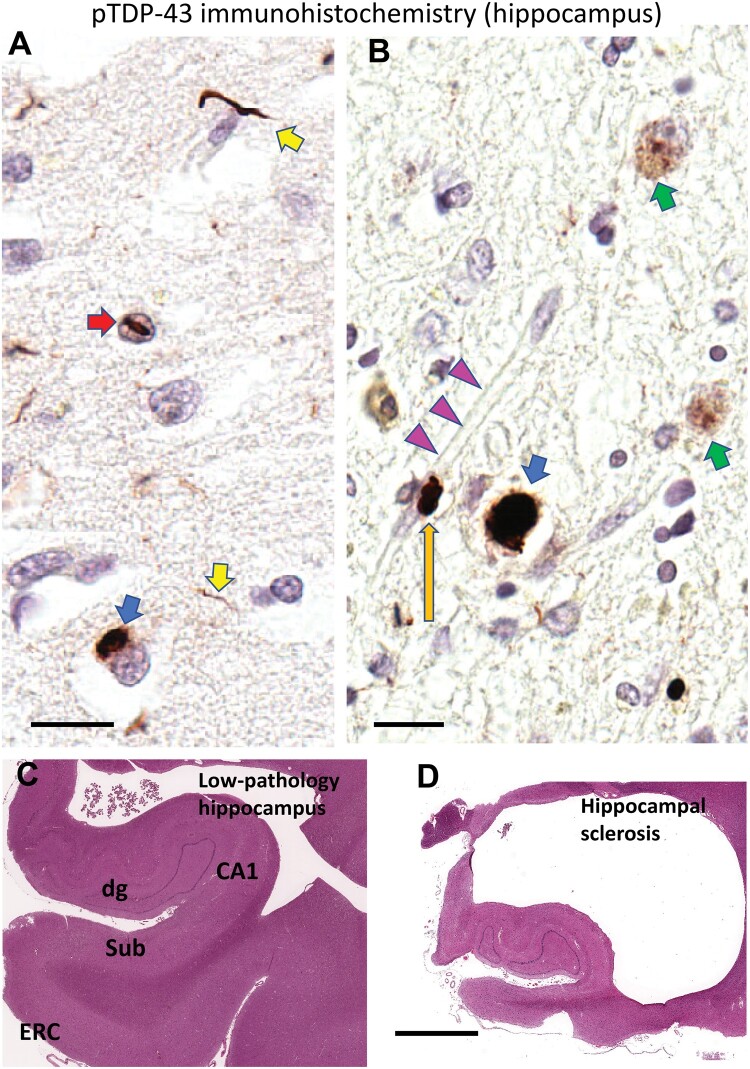
(**A**, **B**) Limbic-predominant age-related TDP-43 encephalopathy neuropathologic changes (LATE-NC) and (**D**) hippocampal sclerosis of aging (HS-A). (**A**, **B**) Sections stained for phosphorylated TDP-43 (pTDP-43) immunohistochemically. The immunostaining signal is brown, nuclei are counterstained blue with hematoxylin. Notable LATE-NC-related features include pTDP-43-immunoreactive neuronal cytoplasmic inclusion (NCI; blue arrow), neurites (yellow arrow), and neuronal intranuclear inclusion (red arrow). (**B**) Early NCIs (green arrow) with punctate staining as well as a more mature and homogeneously stained one (blue arrow). A capillary is indicated with pink arrowheads, with an associated pTDP-43-immunoreactive Lin body (orange arrow). **(C, D)** Hippocampal formations at low magnification, stained with H&E preparation. (**C**) From an individual with minimal hippocampal pathology, for comparison to (**D**), with severe HS-A; note that the scale bars are the same for (C and D), indicating the large amount of atrophy of the hippocampal formation in (D). Scale bars: A, B = 20 µm; C, D = 4 mm. The pTDP-43 antibody used was the 1D3 clone (250) as reported previously (251).

Before discussing associations between LATE-NC and other pathologies and genetics, it is important to address the pathological phenotype itself. TDP-43 proteinopathy (mislocalized, misfolded TDP-43 protein; Fig. 1A, B) was first recognized as a pathological feature in diseases along what is now considered to be a pathological spectrum that includes amyotrophic lateral sclerosis (ALS) and frontotemporal lobar degeneration with TDP-43 inclusions (FTLD-TDP) (20, 21). However, in recent years, it is increasingly recognized that TDP-43 pathology is analogous to tauopathies (22–24). Like Tau pathology, TDP-43 pathology appears to be stimulated by many “upstream” factors, and both pathologies produce many additional “downstream” deleterious effects that include loss of normal functions, and gain of new toxic functions (24–26). Indeed, numerous (more than 20) different TDPopathies have now been identified (24). Recent studies have provided additional insights into the pathogenetic mechanisms of TDP-43 dysregulation, which include exon mis-splicing, aberrant polyadenylation, and various cell type-specific functional impairments (27–32).

From neuropathologic and clinical standpoints, there are relatively clear delineations between LATE-NC and other TDP-43 pathologic entities, including FTLD-TDP (10, 33–36). There are also relatively clear delineations between LATE-NC and other neurodegenerative disease categories, including Tau and α-synuclein pathologies. A minority of cases may fall outside of clear-cut diagnostic groupings. The focus of this review is on common neuropathologies; FTLD-TDP (1:1000 lifetime risk [37, 38]) will not be discussed in depth. Nonetheless, there are known to be unusual TDP-43 pathology cases that fall into diagnostic gray areas and some aspects of classification have been debated (33, 35, 39–42).

The staging system for LATE-NC severity (1), was based on prior work (first from Dr Keith Josephs and colleagues), that identified the stereotypical pattern of TDP-43 pathology in aging brains across a spectrum of disease severity (7, 43). The LATE-NC staging system was intended to be useful for routine neuropathological assessments (1). There are 3 recognized stages, based on the anatomic location and severity of TDP-43 pathology: LATE-NC Stage 1 is characterized by any TDP-43 pathology in the amygdala or hippocampal regions, but not a TDP-43-immunoreactive neuronal cytoplasmic inclusion (NCI) in both; LATE-NC Stage 2 indicates that there is at least 1 NCI in both the amygdala and the hippocampus regions; and, brains with LATE-NC Stage 3 have TDP-43 pathology in the amygdala region, hippocampal region, and middle frontal gyrus (35).

Other TDP-43 pathology staging systems have been developed that distinguish 5 or 6 stages of TDP-43 pathologic distribution in aging (7, 43); the additional stages provided a more complete delineation of TDP-43 pathologic expansion outside the medial temporal lobe, that is between LATE-NC stages 2 and 3. There are several reasons that the 3-stage LATE-NC system was chosen by a consensus panel of experts for routine neuropathologic examination (1): (1) The 3-stage system provides markers for the earliest, most usual, and most severe stages; (2) a higher number of stages would introduce more variability with correspondingly lower inter-rater reliability; (3) there are not compelling reasons to add new stages based on clinical-pathological correlation—that is, more pathological stages would not provide added information for clinicians or families to explain symptoms during life; and (4) additional sampling, sectioning, and staining was discouraged due to economic reasons for neuropathology labs. Despite these benefits of a 3-tier staging scheme for widely used diagnostic purposes, sampling more brain regions involved by TDP-43 pathology, using center-specific protocols, has provided insights in the research context (7, 43, 44).

Assessments of “early LATE-NC” (autopsy series that included minimal pathology cases) indicated that there are differing patterns of presumed incipient TDP-43 pathology in LATE-NC (45, 46). Some brains show early TDP-43 pathology primarily associated with neurons that are vulnerable to Tau pathology (47); these microlesions were termed “tangle-associated TDP-43” (48), partly because of their neurofibrillary tangle (NFT)-like histomorphology and partly because the intracellular inclusions often co-stain using antibodies against both Tau and TDP-43 proteins. A different group of LATE-NC cases show TDP-43 pathology in the medial temporal lobe that partially resembles FTLD-TDP staining patterns (45, 49). Still other cases show both Alzheimer disease neuropathologic change (ADNC)-like and FTLD-like patterns in the same brain (46). (There is poor inter-rater reliability in these diagnostic determinations [36]). It is important to note that despite the differing patterns of TDP-43 pathology, as a rule, in early LATE-NC, the pathological spread through the brain starts in or near the amygdala and then mostly maintains medial temporal lobe predominance (46). Even in the ∼15% of LATE-NC cases that progress to LATE-NC Stage 3, the density of TDP-43 pathology in the neocortex tends to remain considerably lower than that seen in FTLD-TDP (2, 33, 34).

### Alzheimer disease neuropathologic change

**Figure 2.**
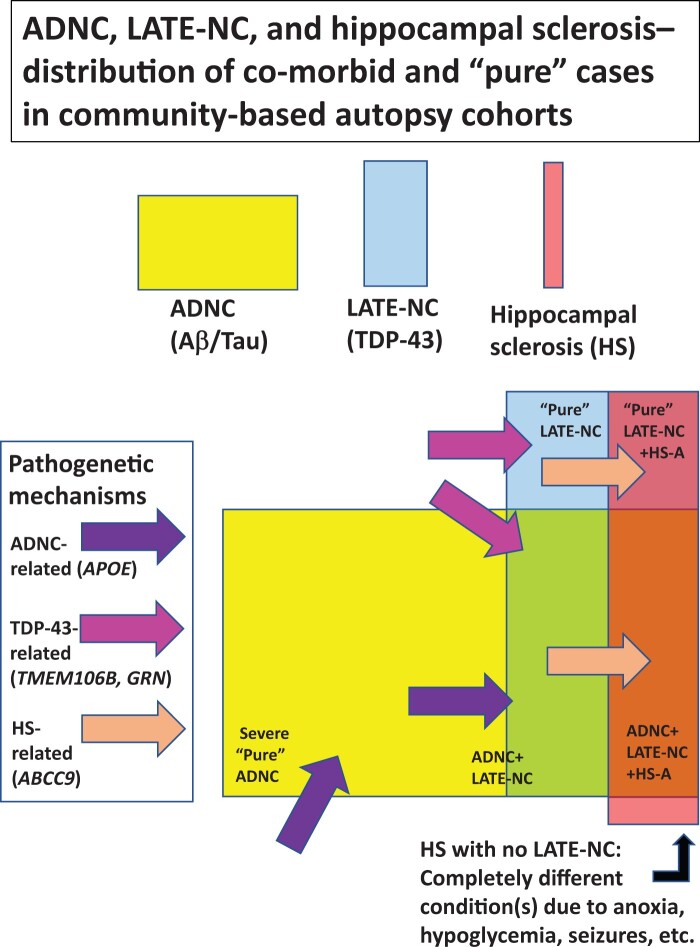
A schematic representation of some of the common pathologies in human populations—how they often overlap and are driven by separate pathogenetic mechanisms. Note that around half of severe Alzheimer disease neuropathologic change (ADNC) cases have comorbid LATE-NC. Somewhat fewer than half of LATE-NC cases have comorbid hippocampal sclerosis of aging (HS-A). Cases with LATE-NC+HS-A lacking ADNC should be considered a subtype of “pure” LATE-NC because it is likely that TDP-43-related mechanisms drive the HS-A pathology. A minority of cases with HS pathology lack LATE-NC; these are associated with other mechanisms and do not map onto the same disease paradigm as HS-A.

The existence of both ADNC and LATE-NC in the same brain at autopsy is common and the combination is impactful according to clinical-pathologic studies (50–52). Having both ADNC and LATE-NC as co-pathologies is associated with a swiftly progressing and severe disease course, with more brain atrophy, in comparison to either LATE-NC or ADNC alone (7, 52–55). Cases with ADNC+LATE-NC also are disproportionately likely to have comorbid Lewy body disease (LBD) (53, 56); this suggests widespread proteinopathy that theoretically may extend beyond Tau, Aβ, TDP-43, and α-synuclein (57). The exact associations between Aβ and LATE-NC are unclear but neuritic amyloid plaques (which tend to contain Tau pathology) are associated with increased TDP-43 pathology in LATE-NC (58). Regardless, approximately 50% of brains with severe ADNC (Braak NFT stages V or VI) have LATE-NC, whereas only ∼25% of cases without any ADNC (i.e. lacking amyloid plaques or advanced Braak NFT stages) have LATE-NC (2). See Figure 2 for a heuristic, visual schematic of ADNC, LATE-NC, and hippocampal sclerosis (HS) according to observations from community-based autopsy cohorts.

“Pathologic synergy” refers to hypothesized interaction(s) between different subtypes of pathology and implies that 1 pathology promotes the other, or that both directly exacerbate each other (59–61). Tauopathy is a subcomponent of ADNC that may synergize most directly with TDP-43 proteinopathy in LATE-NC (47, 60–64). In comparing between Tau and TDP-43 pathologies, both are common but Tau pathology is more common; some degree of Tau pathology is universal by middle age (65). Both Tau NFTs and TDP-43 NCIs are by definition intracellular within neurons, and NFTs and NCIs are often seen in the same neuronal cytoplasm (47, 48, 60, 66). More severe ADNC is associated with higher likelihood of LATE-NC (2, 67), but see (68). Further, the presence of LATE-NC is also associated with more severe (higher Braak NFT stage) primary age-related tauopathy (PART) (2), which lacks amyloid plaques by definition (69). Numerous other dementia-related conditions have been shown to comorbidly express the pathological phenotypes of Tau and TDP-43 proteinopathies (24, 70, 71).

**Figure 3.**
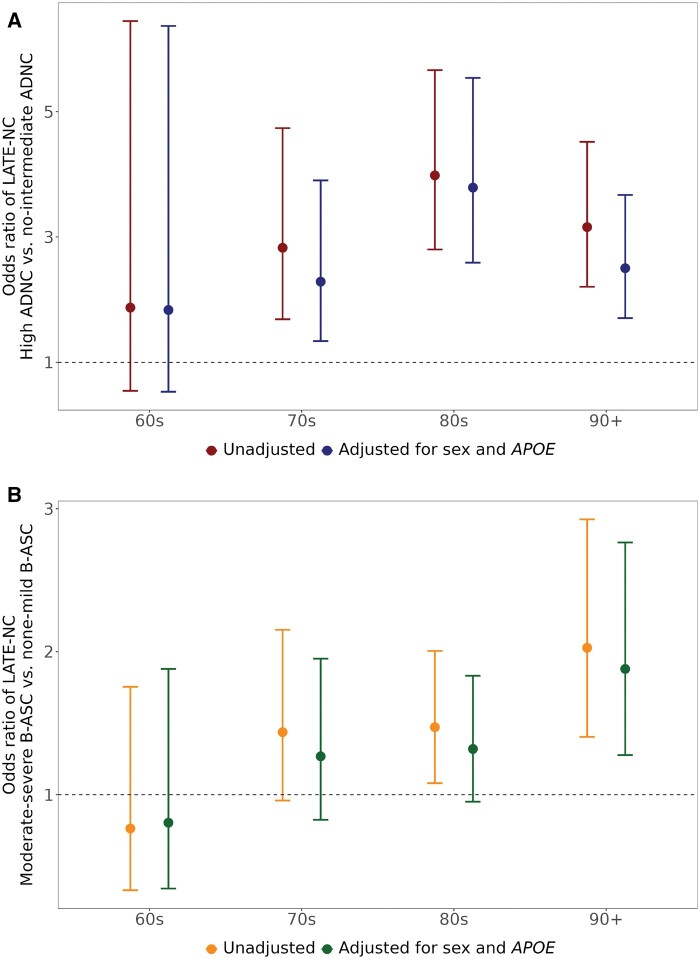
Shown are data from the NACC Neuropathology Data set (n = 2240 cases) using methods as described in detail previously (74) to indicate the associations between LATE-NC with Alzheimer disease neuropathologic change (ADNC; panel **A**) and with brain arteriolosclerosis (B-ASC; panel **B**) across the aging spectrum. In this cohort, ∼25% of cases had LATE-NC. Odds ratios of LATE-NC for high ADNC versus no, low, and intermediate ADNC **(A)** and for moderate and severe B-ASC vs none and mild B-ASC **(B)** estimated by logistic regression models unadjusted (red and orange) and adjusted for sex and the number of ε4 alleles of *APOE* (blue and green).

**Figure 4.**
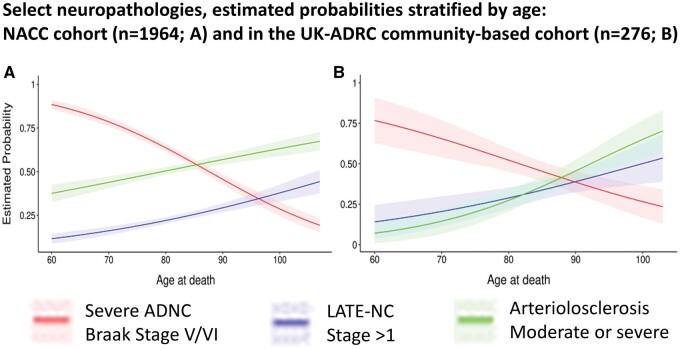
Shown are the same data as in Figure 3 (n = 2240 NACC cases) as described in detail previously (74) to indicate the estimated probabilities of subtypes of pathology, along the human aging spectrum. Since NACC has many clinic-based cohorts that may be less population-representative (252), we split out the cases from the University of Kentucky ADRC (UK-ADRC), which follows a community-based cohort (253). Estimated probabilities with 95% confidence intervals over age at death by logistic regression models for severe ADNC (red), LATE-NC Stage >1 (blue), and moderate-to-severe brain arteriolosclerosis (B-ASC; green) in the NACC cohort excluding the UK-ADRC cohort **(A)** and in the UK-ADRC cohort itself **(B)**. Note that the probability of severe ADNC declines whereas LATE-NC and B-ASC pathologies increase.

The association between ADNC and LATE-NC appears to remain robust across the aging spectrum but it is most pronounced in advanced old age. To convey data related to odds ratios of LATE-NC for high ADNC versus no/low/intermediate ADNC stratified by age at death (categorized into 4 responses) in the NACC Neuropathology Dataset see Figure 3 (72, 73). These data derive from a sample exactly as described in a recent paper using NACC data (74). As in other autopsy cohorts, LATE-NC is relatively likely to occur in cases with high ADNC. The same data were analyzed to convey the estimated probabilities of different neuropathologic findings across the aging spectrum (Fig. 4). Note that LATE-NC maintains an increase in prevalence year-by-year in the oldest-old whereas ADNC severity peaks earlier in the human aging spectrum, as shown previously (1, 74–76).

**Figure 5.**
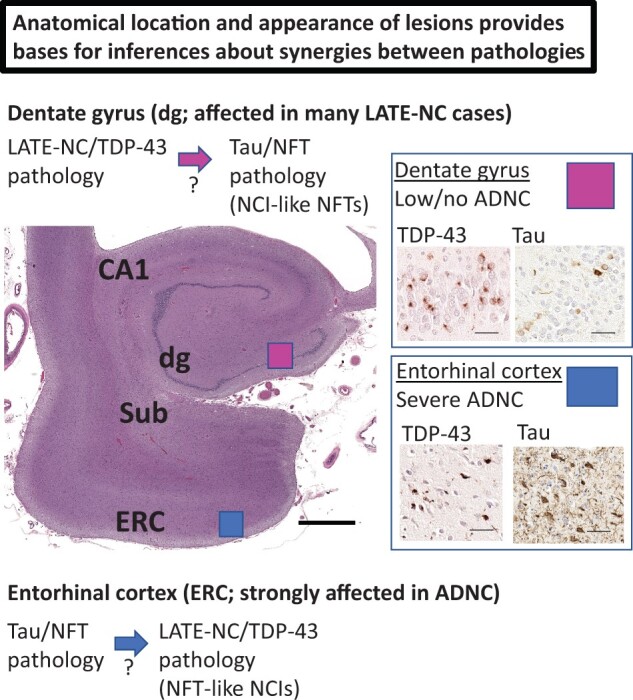
Aspects of pathologic synergy between Tau pathology and TDP-43 pathology may be bidirectional, and distinctive in different anatomical locations. Shown is a coronal section of a hippocampal formation stained with H&E to indicate the anatomic location of the dentate gyrus (dg), CA1, subiculum (Sub), and entorhinal cortex (ERC). The dg commonly shows TDP-43-immunoreactive NCIs in cases with LATE-NC. In these cases, even with minimal Alzheimer’s disease pathology otherwise, one tends to find Tau-immunoreactive NFTs that resemble NCIs histomorphologically. By contrast, the ERC is a location known to be vulnerable to early Tau NFT development. In this region, one may find TDP-43-immunoreactive inclusions that resemble Tau NFTs. These are circumstantial evidences to support the hypothesis that Tau pathology can promote TDP-43 pathology, and vice versa. Scale bars: H&E = 2 mm; dentate gyrus = 30 µm; entorhinal cortex = 40 µm.

There are data in support of the hypothesis that the synergistic associations between Tau pathology and TDP-43 pathology in LATE-NC may be bidirectional in terms of causation, that is each may promote the occurrence of the other. Relevant clues can be gleaned from an analysis of the anatomical distribution of Tau and TDP-43 pathologies in autopsy cohorts. An early anatomical site of involvement of NFTs in ADNC is the entorhinal cortex (77), whereas an early site of involvement of TDP-43 pathology in some LATE-NC cases is the hippocampal dentate gyrus (43, 78). If tauopathy leads to TDP-43 pathology, then one would predict that early in ADNC, there may be colocalization of Tau and TDP-43 pathology in entorhinal cortical neurons vulnerable to NFTs. These “TDP-43 associated tangles” (as mentioned above) have been reported (45, 48, 79). On the other hand, if TDP-43 pathology promotes tauopathy, one would expect “pure LATE-NC” (lacking substantial ADNC) to have Tau-immunoreactive intraneuronal inclusions, colocalized with TDP-43 NCIs, in the dentate granule cells, well before predicted by classic Braak NFT staging (77). This also has been shown to be true (47, 80, 81). A visual depiction of these phenomena is presented in Fig. 5.

Additional reports have emerged from different experimental systems supporting the idea of bidirectional synergies between Tau and TDP-43 pathologies. Since normal TDP-43 function is partly related to transcript splicing regulation, and the cognate Tau-encoding gene (*MAPT*) transcript splicing can be linked to Tau pathology, it is both logical and experimentally verified that TDP-43 pathologic changes may promote Tau dysregulation and pathology (82–84). Further, a *MAPT* mutation has been reported with various comorbid pathologies that include TDP-43 proteinopathy (85). As such, we conclude that there are multiple data in support of the hypothesis that Tau pathology promotes TDP-43 pathology, and vice versa.

### Age-related tau astrogliopathy

**Figure 6.**
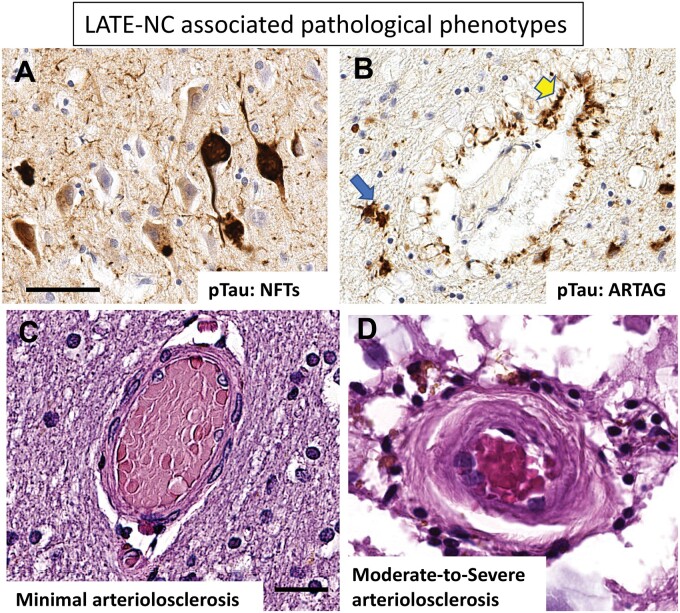
Photomicrographs depict select pathologic features associated with LATE-NC, including Tau NFTs **(A)**, ARTAG **(B)**, and brain arteriolosclerosis **(C, D)**. Panels **A** and **B** shows sections stained for phosphorylated Tau protein with immunohistochemistry, and the photomicrographs were taken at the same magnification. Note that the stained structures in (**A**) depict pathology within neuronal cells, whereas the pathology in (**B**) shows star-shaped astrocytes (blue arrow), and cells and foot processes that surround a small blood vessel (yellow arrow). (**C**) A relatively normal-appearing small arteriole in human brain for comparison to (**D**) that shows a human brain region with moderate-to-severe arteriolosclerosis. Note the rim of inflammatory cells outside the media of the blood vessel (**D**). Scale bars: A, B = 60 µm; C, D = 30 µm. The pTau antibody used was the PHF-1 clone (254) as reported previously (251).

Another subtype of tauopathy associated with LATE-NC is age-related tau astrogliopathy (ARTAG). Unlike NFTs, which by definition are Tau inclusions in neurons, ARTAG is characterized by Tau pathology in astrocytes (86, 87) (Fig. 6). Tau-immunoreactive astrocyte pathologies in ARTAG may display a range of histomorphologic appearances, including “thorn-shaped” and “granular-fuzzy” astrocytes (as visualized with Tau immunohistochemistry) that may be seen in any anatomic area but particularly in subpial, subependymal, and white matter regions (88–90). ARTAG is likely to occur in brains with comorbid TDP-43 pathologies, including LATE-NC (91–94). For example, Forrest et al showed that ARTAG, and a novel Tau pathology termed age-related tau oligodendrogliopathy (ARTOG), were associated with LATE-NC (94). Sordo et al (74) and Katsumata et al (92) confirmed that ARTAG was associated with LATE-NC. Yokota et al (95) reported that the presence of amygdala granular-fuzzy astrocytes was associated with LATE-NC as well as with Tau-immunoreactive argyrophilic grain pathology. Notably, like some other pathologies discussed herein, ARTAG is strongly associated with advanced age, but unlike some others, is more prevalent in males (74, 96).

The mechanisms underlying the pathological synergies or common causes shared by TDP-43 pathology and ARTAG are currently poorly understood. Upstream factors such as traumatic brain injury (TBI) and cerebrovascular pathologies may stimulate both TDP-43 pathology and ARTAG (74, 97, 98). However, there may be actual synergy, wherein TDP-43 dysregulation promotes ARTAG and/or vice versa. Some related insights may be learned by studying rare conditions. For example, a *TARDBP* mutation was associated with a pathological phenotype that included ARTAG-like glial tau pathology (99). Further, a case was identified with *GBE1* mutations leading to both ARTAG-like and TDP-43 pathologies (100). Another FTLD-linked *GRN* mutation also was correlated with both ARTAG-like and TDP-43 pathologies (101). In a larger autopsy series, ARTAG was relatively common in brains affected by FTLD-TDP (63). These observations again underscore the synergistic interactions and comorbidity of Tau and TDP-43 pathologies, affecting astrocytes as well as other cell types in the brain.

### Hippocampal sclerosis (HS) of aging (HS-A)

HS was recognized as a neuropathologic feature associated with dementia in 1994 (102), and the association between HS and TDP-43 proteinopathy was first reported in 2007 (103); both of these advances were accomplished by Dr Dennis Dickson and colleagues at the Mayo Clinic (Fig. 1). Other researchers have helped to gain further insights into the clinical, pathological, and radiographical features of LATE-NC with comorbid HS (104–114). It is also notable that, when multiple variable regression-based statistical models are applied, factoring in TDP-43 proteinopathy and other pathologies, HS is associated with substantial independent cognitive impairment (1, 3, 4, 78, 115–118).

Although the presence of HS heralds an impactful disease process in a clinical sense, the basic definition of HS, and even the term itself, are problematic. According to the consensus-based definition, HS refers to “pyramidal cell loss and gliosis in CA1 and subiculum of the hippocampal formation that is out of proportion to AD neuropathologic change in the same structures” (119). This captures key aspects of the pathology but unfortunately this definition is highly nonspecific and, given the histopathological variability in actual practice, individual neuropathologists must apply their own ad hoc criteria for the many cases that have intermediate levels of cell loss and gliosis (120). It also is widely known that a number of different diseases produce a histopathologic picture that meets the definition. For example, the clinical literature usually refers to a seizure disorder when applying the term HS, and pathology that is labeled HS is also seen in completely different circumstances related to brains affected by anoxia, hypoglycemia, and various infections that target the hippocampus (121–123). We and others have proposed a term that is more specific for HS in the context of LATE-NC, which is “hippocampal sclerosis of aging,” or HS-A (92, 113, 124). The term HS-A is useful and we will apply it subsequently in this present review but it has the drawbacks of not being consensus-based and also is still not specifically defined in terms of the precise histopathologic features other than LATE-NC, cell loss, and gliosis in the hippocampal formation. More rigorous and standardized terminology will be required in the future.

Despite the uncertainties, HS-A is a research focus where data-driven, causal pathoetiologic inferences can be made. This is because the distribution of pathologic findings in multiple autopsy cohorts are relatively consistent and instructive: the great majority of cases with HS in aging have LATE-NC; LATE-NC is approximately twice as common as HS-A; and brains with unilateral HS-A generally have bilateral TDP-43 proteinopathy (75, 78, 111–113, 125–129). These observations lead to the following corollary conclusions: HS-A occurs “downstream” of LATE-NC, and thus LATE-NC-related processes are the major drivers of HS in aging. Given the nonspecific implications of the term HS, the extant data strongly imply that LATE-NC is not only a co-pathology with HS-A. Instead, LATE-NC + HS-A is best considered a severe manifestation or subset of “pure LATE-NC,” with HS-A in LATE-NC analogous to loss of pigmented neurons in Parkinson disease substantia nigra.

Many unanswered questions about HS-A remain. For example, why do only a subset of cases with LATE-NC have HS-A? And, is HS-A influenced by (caused by, or synergistic with) ARTAG and/or cerebrovascular disease? Given that HS in other contexts is associated with vasculopathy and/or ischemia-reperfusion (121), and astrocytes are integral components of the blood-brain barrier, the potential seems high for HS to be exacerbated by astrocyte dysfunction. In animal models, TDP-43 perturbations lead to robust astrocytosis, inflammation, and blood-brain barrier dysfunction (30, 130, 131). Recently, we showed in human brains that hippocampal astrocytosis in LATE-NC+HS-A was more severe than that of ADNC, LATE-NC, or HS alone (132). These data indicate that LATE-NC+HS-A may be tied to TDP-43 dysregulation, astrocytic signaling, blood-brain barrier dysfunction, and/or neuroinflammation.

### Cerebrovascular pathology, particularly brain arteriolosclerosis (B-ASC)

Another category of brain pathology that has been associated with LATE-NC is cerebrovascular disease. An early study described cerebrovascular pathologies in a cohort of 13 individuals with HS and dementia (102). Ensuing autopsy series also showed enrichment for cerebrovascular pathology in brains with LATE-NC and HS (Table 1). The subtype of cerebrovascular pathology most often associated with LATE-NC + HS-A was B-ASC, characterized by thickened and/or dysmorphic arterioles in the brain (133) (Fig. 6).

**Table 1.** Published studies that evaluated the association between cerebrovascular pathologies and LATE-NC/hippocampal sclerosis (HS) phenotypes

Ref.	Year	Pheno-type(s)		Associated with LATE-NC and/or HS?						
			Sample size (total)	Arterio-loscler-osis	Athero-scler-osis	Micro-infarcts	Lacunar infarcts	Large infarcts	CAA	Note
(102)	1994	HS	13	Overall high level of cerebrovascular pathologies in HS case series						No control group or type-specific associations
(106)	2002	HS	48	“…medical record review indicated higher frequencies of clinical stroke and neuroradiologic white matter abnormalities in the HS group”						HS pathology was not associated with vascular neuropathology phenotypes
(133)	2014	HS	2218	Yes	No	No	No	No	No	Study included digital pathology methods of vessel geometry in a subset; also included NACC NP data.
(235)	2018	LATE-NC	929	Yes	No	No	No	No	No	NACC study focusing on TDP-43 pathology yes/no
(236)	2020	HS and LATE-NC	749	Yes (post. water-shed only)	No	No	N/A	No	Yes	Rush U./ROS-MAP study. Only posterior watershed brain arteriolosclerosis was associated with LATE-NC
(237)	2020	LATE-NC	616	Yes	No	No	No	No	No	NACC study focusing on TDP-43 pathology yes/no
(128)	2020	HS and LATE-NC	359	N/A	Yes (with HS)	(Infarct groups clustered together) Not associated with either HS or LATE-NC			N/A	U. Pitt ADRC study; HS but not LATE-NC associated with atherosclerosis.
(238)	2021	HS and LATE-NC	98	Yes	No	No	N/A	N/A	No	Duke/UNC ADRC study; HS and LATE-NC associated with brain arteriolosclerosis
(239)	2021	HS	122	HS was associated LATE-NC and with “vascular changes in the basal ganglia” but not with large infarcts or CAA or ADNC						U. Antwerp cohort, focusing on FTLD and vascular pathology system of Deramecourt (138) was used
(240)	2022	HS and LATE-NC	203	Yes	No	No	N/A	N/A	No	Duke/UNC ADRC study; LATE-NC associated with brain arteriolosclerosis in young-old and older-old
(92)	2023	HS and LATE-NC	408	Yes	No	“MVL”; No	N/A	N/A	No	UCI 90+Study; readouts here relate to quantitative assessments of HS, not the dichotomous HS± ones.
(241)	2023	HS and LATE-NC	4354	No	No	No	No	No	No	International multicenter study with disparate brain arteriolosclerosis scoring methods used across centers

ADRC, AD Research Center; CAA, cerebral amyloid angiopathy; MVL, microvascular lesions; N/A, data not available; ROS-MAP, Rush Religious Order Study/Memory-Aging Project; UCI, University of California Irvine; UNC, University of North Carolina; U.Pitt, University of Pittsburgh (Other abbreviations defined elsewhere.)

Like most of the pathological features discussed in this review article, B-ASC is at present incompletely understood, inexactly defined, and not always diagnosed using standardized methods (134–136). This adds a potentially significant confounding factor to scientific inquiry, particularly when trying to integrate the results from cohorts that have divergent operationalization of B-ASC. Readers are referred to prior reviews (134, 137–140). Although B-ASC is associated with diabetes and hypertension (particularly in persons younger than age 80 at death) and B-ASC is also associated with HS, HS has not been shown to be associated with either diabetes or hypertension (117, 133, 134, 136, 141). These data indicate that the pathological phenotype described as B-ASC that is driven by hypertension and/or diabetes is nonidentical (or completely different) in comparison to that which is associated with LATE-NC/HS-A. For the sake of the present article, the term B-ASC should be considered a proxy label for a subset of brain microvasculopathy that is currently in its early stages of being understood. Figure 3B provides a depiction of LATE-NC odds ratios for moderate/severe B-ASC versus none/mild B-ASC stratified by the 4 age at death categories in the NACC Neuropathology Dataset (72, 73).

The neurovascular unit (NVU), which appears to be disrupted in B-ASC (134, 135), comprises a number of different cell types that together regulate blood flow, filter and exchange substances between blood and brain, modulate neuroinflammation, and perform other functions (142, 143). Correspondingly, the influence of TDP-43 pathology may be exerted on behalf of multiple NVU cell types that include astrocytes, endothelial cells, and/or vascular mural cells, that is pericytes and smooth muscle cells. A subset of TDP-43 proteinopathy has been reported within astrocyte foot processes near capillaries (144). These TDP-43-immunoreactive structures were termed Lin bodies (Fig. 1B) and their influence on vascular function is unknown (35, 144). More work is required to characterize the presence and impact TDP-43 pathology that takes place within glial cells in LATE-NC and other conditions. Otherwise, astrocytes in LATE-NC were discussed above in the section related to ARTAG.

Intriguing recent research focusing on endothelial cells has uncovered another mechanism whereby TDP-43 dysregulation may lead to vasculopathy (and/or vice versa). Endothelial cell TDP-43 knockdown or dysregulation in animal models led to impaired endothelial function and attenuated angiogenesis (30, 145, 146). These influences could cascade to other cell types and promote broader levels of dysfunction in LATE-NC. This would lead to increased inflammation, secondary neurodegeneration, and/or impairment of mechanisms of resilience. Since endothelial cells are often involved in cerebral amyloid angiopathy (CAA) (147, 148), it is of interest that most prior studies do not find an association between CAA and LATE-NC (Table 1).

In addition to affecting astrocytes and endothelial cells, LATE-NC may also have deleterious impacts on the NVU’s mural (blood vessel wall) cells. Using microvessel extracts from the parietal cortex of participants of the Rush University Religious Orders Study (149), in a Western blotting analysis, Bourassa et al (150) found that levels of mural markers referent to smooth muscle or pericyte cells were correlated with TDP-43 pathology and protein cleavage products. There is also support for a hypothetical mechanism linking NVU mural cell dysfunction with HS-A through a genetically inherited mechanism, as described below.

Another potentially disease-driving mechanism, although mostly beyond the purview of the current article, is senescence. The prevalence of B-ASC (like LATE-NC, HS-A, and ARTAG), increases with every added year in advanced old age (74, 75, 134, 151, 152). By contrast, the prevalence of pathologies such as ADNC, FTLD-TDP, and others level off and decrease in advanced old age (38, 76, 152). The association between B-ASC and LATE-NC is stronger in the “oldest-old” (Fig. 3B). Note that, whereas Figure 4 shows the proportion with moderate-severe B-ASC, over 80% of individuals beyond age 80 had some identifiable B-ASC (141, 153, 154). Small-vessel pathologic changes have been shown to be a component of senescence, across multiple organs and organisms (155–160). However, the LATE-NC-relevant senescence-driven B-ASC mechanisms are poorly understood and data from other contexts may not correlate perfectly with human brain-specific mechanisms.

### Other conditions that may influence and/or resemble the brain changes of LATE-NC

Two additional pathological paradigms with aspects of overlap with LATE-NC are LBDs and the brain changes that are associated with repetitive TBI—chronic traumatic encephalopathy (CTE). The term LBDs indicates the presence of inclusion bodies in neurons and/or glia comprising misfolded α-synuclein. There are several notable phenomena at the nexus of LATE-NC and LBDs. Given the likelihood of each occurring in isolation, a disproportionately large proportion of brains have a combination of pathologies that include LATE-NC, ADNC, and Lewy bodies (52). This “quadruple misfolded protein” (Aβ, Tau, TDP-43, and α-synuclein pathologies in the same brain) phenotype is characterized by a swift and severe dementia syndrome, often with clinical psychotic symptoms (52, 53). The prevalence of this pattern of pathologies indicates that polyproteinopathy-influencing pathways of pathogenesis exist that are imperfectly understood (52, 53, 161). The amygdala has been noted to be an anatomical region where LBD as well as TDP-43 pathology are often involved early in pathogenesis (59, 117). In cases with early LATE-NC that also have amygdala LBD co-pathology, the TDP-43 pathology pattern tends to be different from that seen in cases lacking Lewy bodies (46, 162).

CTE is another clinical-pathological entity with aspects of overlap with LATE-NC. While the pathognomonic features of CTE involve intraneuronal Tau pathology (163, 164), TDP-43 pathology may exist as a co-pathology, even among individuals age 50 and younger. The TDP-43-like pathology in CTE may be localized to the hippocampus, but often it occurs in a brain region that tracks very separately from the stereotypical LATE-NC stages (165) and other TDP-43 pathologic staging systems (7, 43). Intriguingly, CTE may also show HS pathology, even among individuals of an age group (persons younger than age 60 years) who otherwise would be unlikely to have HS-A (165).

**Figure 7.**
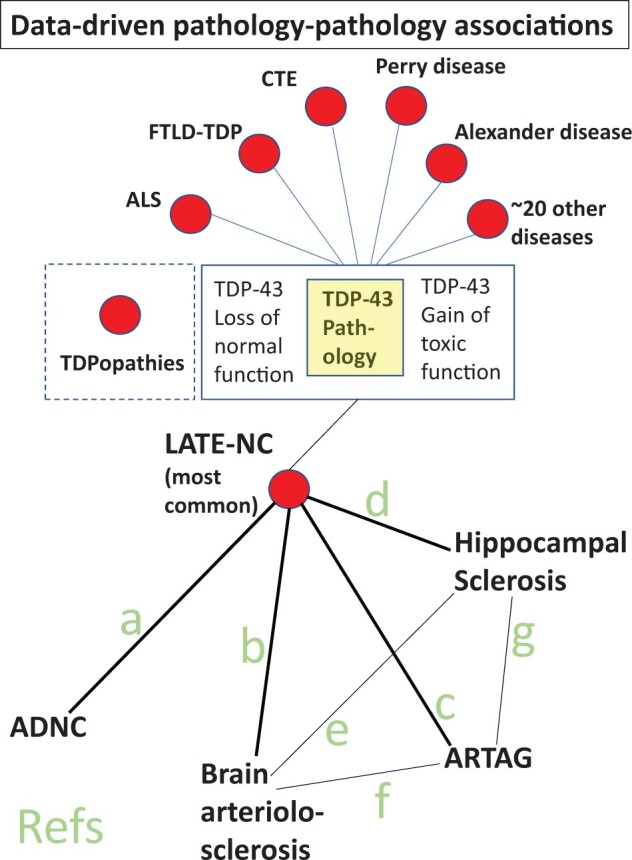
Schematic depiction of TDPopathies (diseases with TDP-43 pathology) including LATE-NC which is most common by far, along with LATE-NC-associated neuropathologic phenotypes. References for the studies that reported these associations shown as lines between subsets of pathology: (a) Refs. (2, 103, 255); (b) Refs. (235, 236, 238); (c) Refs. (74, 94, 95); (d) Refs. (103, 110); (e) Refs. (103, 110); (f) Ref. (74); (g) Ref. (92).

Collectively, the multitude of pathologies with overlapping features provides important clues about how homeostatic or deleterious influences in different brain conditions are linked to healthy and disease states. These phenomena also indicate the challenges of clinical-pathological correlation when an incomplete list of neuropathologies are factored in to statistical models—1 pathologic feature may actually be a proxy measure for others. See Figure 7 for a schematic presentation of prior work, including pathology-pathology associations and selected references. So far, we have highlighted the pathologic manifestations that are associated with cognitive impairment. These data help to contextualize parallel studies of upstream factors that influence cognitive impairment or resilience.

## GENETIC ASSOCIATIONS WITH LATE-NC AND HS-A

Genetic studies provide critical insights into disease-related mechanisms. To date, 4 genes have been reported (with replication) to harbor risk alleles associated with LATE-NC: Granulin (*GRN*), Transmembrane protein 106B (*TMEM106B*), Apolipoprotein E (*APOE*), and Sortilin related receptor 1 *(SORL1*) (111, 166–172). We will discuss separately the association between HS-A and ATP-binding cassette sub-family member 9 (*ABCC9*) genetic variants. There are a number of other genes that have demonstrated suggestive or otherwise not-yet replicated associations with LATE-NC/HS phenotypes (Table 2).

**Table 2.** Genes linked to LATE-NC/hippocampal sclerosis (HS) neuropathologic phenotypes

Main finding replicated?	Gene (protein)	Disease-related phenotype(s)	Notes	Selected Refs.
Replicated	*TMEM106B*	LATE-NC, FTLD-TDP, pediatric leukodystrophy, viral pathogenesis	TMEM106B C-terminal amyloidogenic fragments form disease-associated “inclusion bodies”	(129, 171, 173, 188, 242, 243)
	*GRN (*Progranulin*)*	LATE-NC, HS, FTLD-TDP, neuronal ceroid lipofuscinosis	GRN is an inflammation-regulating growth factor also implicated in cancers	(167, 170, 244, 245)
	*APOE*	LATE-NC, ADNC, Lewy body diseases, hypercholesterolemia	*APOE* is prime driver of late-onset AD neuropathologic changes	(111, 129, 171, 205, 236, 246)
	*SORL1*	LATE-NC+ADNC, clinical AD, clinical FTLD	*SORL1* variants are associated with ADNC+LATE-NC pathologic phenotype	(211, 213, 214, 228)
	*ABCC9 (*SUR2*)*	HS, Cantu syndrome, *ABCC9*-related intellectual disability and myopathy syndrome, cardiomyopathy and cardiac arrhythmias	*ABCC9* variants are also associated with vascular malformations and white matter hyperintensities	(129, 169, 170, 217, 247)
Not yet replicated	*KCNMB2*	HS, possibly ALS, Epilepsy	*KCNMB2* encodes a potassium channel expressed in hippocampus	(129, 166, 230, 231, 248)
	*WWOX*	LATE-NC, HS, arteriolosclerosis, clinical AD, spinocerebellar ataxia, epilepsy	*WWOX* was a GWAS “hit” for clinical AD and pathological LATE/HS	(169, 225, 226, 229, 249)
	*AHRGEF28 (*RGNEF*)*	LATE-NC (suggestive), Cri-du-Chat syndrome	ALS lesions have been shown to stain for RGNEF	(233), in press
	*TPCN1*	LATE-NC (suggestive), clinical AD	TPCN1 is a lysosomal protein linked by GWAS to clinical AD	(213, 228)

Genetic variants in *GRN* and *TMEM106B* were initially demonstrated to be associated with the risk of FTLD-TDP (173–176). These same genes have now also been shown to have genetic variation associated with risk of LATE-NC and HS-A. The *GRN* and *TMEM106B* risk-associated alleles exhibit moderate effects on risk/protection. These phenomena provide compelling evidence for partial pathogenetic overlap between FTLD-TDP and LATE-NC.

As a gene containing variants with common and high-impact risk alleles, *TMEM106B* has a large influence on public health. Aoki et al (168) reported that the frequency of the C-allele of *TMEM106B* single-nucleotide polymorphism (SNP) rs1990622 in HS/HS-A was lower than that in non-HS controls. There was further replication of increased risk for HS-A associated with each copy of the T-allele of *TMEM106B* rs1990622 (169, 170, 177, 178). In linkage with this *TMEM106B* risk-associated SNP, a large haplotype (a set of continuous alleles, inherited together) that incorporates many introns of the *TMEM106B* gene was associated with differential expression of TMEM106B protein (179, 180). Further, the *TMEM106B* gene appears to be pleiotropic for multiple diseases (181–184), and the LATE-NC risk allele in *TMEM106B* may influence healthy brain aging (179, 185). Recently published data from Dr. Rosa Rademakers and colleagues have demonstrated that a carboxy-terminal fragment (CTF) of the TMEM106B polypeptide (amino acid residues 120–254) forms amyloidogenic fibrils that can be visualized with immunohistochemistry as intracellular aggregates in many aged human brains (186–188). The amount of TMEM106B CTF fibrillary aggregates was correlated with the risk allele (they were found in both FTLD-TDP and LATE-NC cases) and they may be highly relevant to LATE-NC pathogenesis, at least in some cases (186, 189–191).

With regard to *GRN*, HS/HS-A risk in aged individuals was associated with the T-allele of the *GRN* SNP rs5848 (167, 171). The same genetic risk variant has been associated with increased inflammatory mediators (e.g. AXL and CLU) in cerebrospinal fluid (192). Separate studies have found that *GRN* gene products (progranulin/granulins) are key mediators in inflammation and wound repair (193, 194). Further, the proteins coded for by both *TMEM106B* and *GRN* have been shown to play important roles in endosomal/lysosomal biology, and there is experimental evidence for functional interaction of these proteins (180, 195–200).

Both risk alleles from the *TMEM106B* and *GRN* genes are differentially represented when comparing populations of African and European ancestries: in persons with European ancestry, the *TMEM106B* risk-associated allele is most frequent (thus, more European-ancestry individuals harbor risk genotypes than not), whereas in persons of African ancestry the *TMEM106B* risk allele is distinctly less frequent, suggesting differing local genetic architectures (201). By contrast, the risk allele for the *GRN* SNP rs5848 is far more common in persons with African ancestry than in persons of European ancestry (201). Coupled with the substantial associative impact of these alleles in terms of disease risk (129), these observations suggest that future therapeutic strategies for LATE-NC and FTLD-TDP may have some different outcomes based on the patient populations that are being served.

In addition to *TMEM106B* and *GRN*, the *APOE* ε4 allele also has been associated with increased risk for LATE-NC (172, 202–205). Not all studies found evidence of a direct association between *APOE* genotype and risk for LATE-NC or HS-A (75, 106, 111, 125, 206). Few individuals with the *APOE* ε4 allele survive into advanced old age without any Aβ plaques (207, 208), and it remains to be seen exactly how the *APOE* genotype promotes TDP-43 proteinopathy (there may be multiple pathways involved). Nevertheless, the strong ADNC-driving influence of *APOE* genotypes is well known, and, along with the pathologic observations described above, these data underscore that brain changes in ADNC, like FTLD-TDP, ARTAG, B-ASC, and CTE, have molecular mechanisms that overlap with LATE-NC.

Another pathogenetic clue comes from the associations between *SORL1* and the ADNC+LATE-NC phenotype. *SORL1* genetic variants were previously linked to AD risk and the SORL1 protein is involved in amyloid-β clearance and endosomal processing of proteins, so it has a highly credible association with ADNC (209–211). *SORL1* mutations have also been linked to FTLD (212). We found an association signal between *SORL1* variation and LATE-NC (213); more specifically, the *SORL1* variant(s) may drive a subtype of LATE-NC that tends to present with comorbid ADNC. Consistent with that hypothesis, a post-hoc analysis indicated that the rs74685827 *SORL1* risk allele was associated with the ADNC+LATE-NC phenotype (213). A separate group of investigators discovered a different genetic variant that was linked to a family lineage with clinical dementia and ADNC+LATE-NC co-pathologies (214).

Genetics have also provided early insights into why a subset of cases with LATE-NC are susceptible to developing comorbid HS-A. The *ABCC9* gene association with LATE-NC/HS-A was discovered via genome-wide association study (GWAS) (169). The results of subsequent studies were variable and 1 study failed to replicate the association between *ABCC9* and either LATE-NC or HS-A (215). Nonetheless, there has been replication; the finding of the associations between *ABCC9* gene variants and HS-A (170), and MRI detected brain atrophy (216), were reported in separate samples from the initial GWAS (169). The gene product of *ABCC9*, the sulfonylurea receptor type 2 (SUR2) protein, is a component of an ATP-sensitive potassium (“KATP”) channel (217). KATP channels serve as metabolic sensors that regulate blood flow that also constitute a molecular substrate for ischemic preconditioning (217–219). The *ABCC9* risk genotype also implicates thyroid hormone dysregulation in LATE-NC (220). When both LATE-NC and HS-A outcomes were included in a statistical model, the *ABCC9* alleles that were associated with altered gene expression of *ABCC9*/SUR2 (i.e. were expression quantitative trait loci [eQTLs]) were associated with risk for HS-A, but not specifically LATE-NC (129). It is also notable that *ABCC9*/SUR2 is known to be highly expressed in pericytes, which are critical mural cells in vascular signaling (217, 221, 222). HS pathology is associated with vasculopathy even in cases lacking LATE-NC (223, 224). Overall, the existing data support the hypothesis that genetic variation at *ABCC9* may render some individuals with LATE-NC vulnerable (or resistant) to HS-A.

In addition to the above genetic loci that have been replicated in relation to their association with LATE-NC/HS-A risk, there have been a number of additional genes and loci with suggestive (but not yet replicated) associations with LATE-NC/HS-A pathologic phenotypes (Table 2). Here, we highlight 2 such genetic loci: *WWOX* (WW Domain Containing Oxidoreductase), and *ARHGEF 28* (Rho guanine nucleotide exchange factor 28)/RGNEF. The *WWOX* gene is on human chromosome 16 and the WWOX protein plays roles in transcription regulation, glucose metabolism, and central nervous system development (225). In a GWAS of clinically assessed AD-type dementia, a SNP in the *WWOX* locus, rs62039712, was associated with clinical AD (226). Subsequently, this variant was not found to be an eQTL in brain tissues, and later AD dementia-related GWAS did not identify *WWOX* as a dementia risk gene (227, 228). In a separate GWAS using HS as a phenotype, *WWOX* returned several suggestive loci, but none were statistically significant after correcting for multiple comparisons (169). However, in a later gene-focused study, several different *WWOX* genetic variants again showed associations with LATE-NC, HS, and/or brain arteriolosclerosis (229). These associations remained largely unchanged after adjustment for ADNC, suggesting that these associations were independent of ADNC (229). Another gene related to LATE-NC/HS-A risk is *ARGHEF28*, which encodes the RGNEF protein. *ARHGEF28* has been reported as a putative ALS gene (230, 231). Along the same lines, its cognate protein RGNEF has been found to localize to hallmark TDP-43-immunoreactive inclusion bodies in ALS patient spinal cord motor neurons (232, 233). We recently found that the *ARHGEF28* SNP rs80190672 was associated with LATE-NC (234). This genotype-phenotype correlation may provide another point of etiologic connection between LATE-NC and the ALS/FTLD disease continuum.

Genetics discoveries to date pertinent to LATE-NC/HS-A should be considered the “low-hanging fruit” that are mostly referent to European-ancestry cohorts. More work is required to elucidate additional associations between genetic factors and LATE-NC/HS-A risk. This research is challenged by the requirement for larger cohorts, broader genotyping (evaluating genetic phenomena beyond SNPs), high-quality neuropathology data, old-age research volunteers, and ethnically and racially diverse samples comprising a broad range of exposures and experiences. Study design improvements in these areas will no doubt enable future discoveries. This research has the potential to aid precision-based health care and perhaps to help guide clinical trial candidacy.

## CONCLUSION

**Figure 8.**
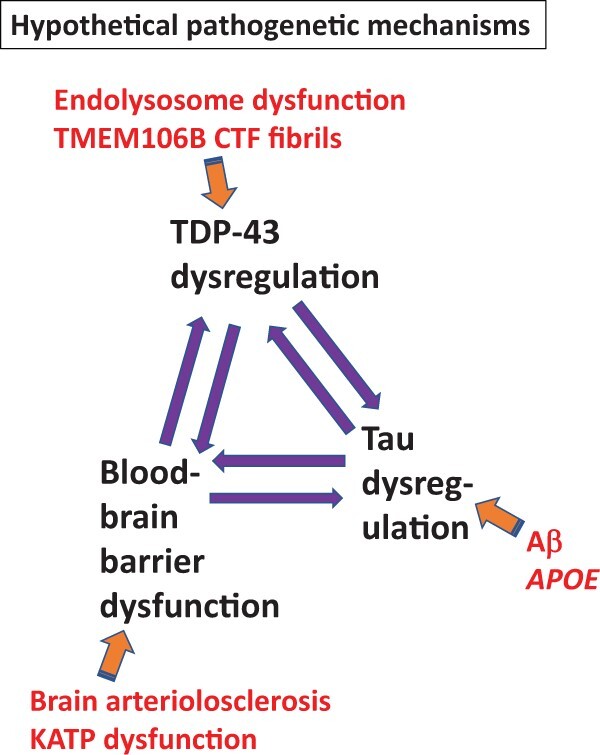
A review of human phenomenology (pathologic and genetics studies) provides preliminary clues about inter-relationships between pathology-driving factors in LATE-NC. TDP-43 pathology is the disease-defining feature of LATE-NC, and often is accompanied by several co-pathologies and/or predicted by genetic risk factors. More specifically, LATE-NC tends to be correlated with Tau pathology and blood-brain barrier dysfunction, which may play added roles in terms of pathologic and, ultimately, clinical manifestations.

Because LATE-NC is associated with different comorbid neuropathologies and with a set of intriguing genetic risk factors, a review of findings from human autopsy studies provides the bases for generating hypotheses about LATE-NC pathogenesis. The patterns of co-pathologies in multiple autopsy series highlight the possible bidirectional importance of TDP-43 pathology along with Tau pathology and B-ASC/vasculopathy. Genetics studies indicate the importance of endolysosomal pathways, ADNC pathways, TMEM106B CTF inclusions, and potassium (“KATP”) channels. A preliminary mechanistic schema is shown in Figure 8. Implications of these data may be far-reaching and clinically relevant. The partial success of anti-Aβ immunotherapies in slowing the progress of ADNC-driven dementia provides new urgency for sharpening our understanding of the underlying mechanisms, and, ultimately, the potential for therapeutic remedies related to non-Aβ contributions to dementia.
